# A new role for P2X_4_ receptors as modulators of lung surfactant secretion

**DOI:** 10.3389/fncel.2013.00171

**Published:** 2013-10-08

**Authors:** Pika Miklavc, Kristin E. Thompson, Manfred Frick

**Affiliations:** Institute of General Physiology, University of UlmUlm, Germany

**Keywords:** P2X_4_ receptor, lamellar body, alveolar epithelial cell, exocytosis, calcium, cellular secretion, pulmonary surfactant

## Abstract

In recent years, P2X receptors have attracted increasing attention as regulators of exocytosis and cellular secretion. In various cell types, P2X receptors have been found to stimulate vesicle exocytosis directly via Ca^2+^ influx and elevation of the intracellular Ca^2+^ concentration. Recently, a new role for P2X_4_ receptors as regulators of secretion emerged. Exocytosis of lamellar bodies (LBs), large storage organelles for lung surfactant, results in a local, fusion-activated Ca^2+^ entry (FACE) in alveolar type II epithelial cells. FACE is mediated via P2X_4_ receptors that are located on the limiting membrane of LBs and inserted into the plasma membrane upon exocytosis of LBs. The localized Ca^2+^ influx at the site of vesicle fusion promotes fusion pore expansion and facilitates surfactant release. In addition, this inward-rectifying cation current across P2X_4_ receptors mediates fluid resorption from lung alveoli. It is hypothesized that the concomitant reduction in the alveolar lining fluid facilitates insertion of surfactant into the air–liquid interphase thereby “activating” it. These findings constitute a novel role for P2X_4_ receptors in regulating vesicle content secretion as modulators of the secretory output during the exocytic post-fusion phase.

## INTRODUCTION

In recent years, P2X receptors have attracted increasing attention as regulators of exocytosis and cellular secretion in a wide variety of organs including the lungs (Burnstock et al., 2012). P2X receptors are membrane cation channels that are activated by extracellular adenosine triphosphate (ATP), the molecular and functional properties of which have been reviewed in detail elsewhere (Surprenant, 1996; North, 2002; Khakh and North, 2006; Burnstock and Kennedy, 2011; Kaczmarek-Hajek et al., 2012). ATP has been known to stimulate cellular secretion for several decades (Rodriguez Candela and Garcia-Fernandez, 1963; Diamant and Kruger, 1967). One of the earliest indications for involvement of P2X receptors in stimulating secretion came from the studies of Cockcroft and Gomperts (1979a,b, 1980). They found that ATP triggers degranulation and histamine release in mast cells via activation of P_2Z_ (Cockcroft and Gomperts, 1980), which later turned out to be P2X_7_ (Surprenant et al., 1996). Since the first cloning of P2X receptor subunits in 1994 (Brake et al., 1994; Valera et al., 1994), P2X receptors have been found to stimulate and modulate various cellular secretion pathways, including fluid secretion in exocrine glands and epithelia (Novak, 2011), secretion of cytokines via release of plasma-derived microvesicles (Solini et al., 1999; MacKenzie et al., 2001) or exosomes (Qu et al., 2007; Qu and Dubyak, 2009).

Moreover, several members of the P2X family have been implicated in regulating exocytosis of secretory organelles in a variety of cell types (Gu and MacDermott, 1997; Ulmann et al., 2008; Jacques-Silva et al., 2010; Gutierrez-Martin et al., 2011; Huang et al., 2011). Substantial evidence suggests that P2X receptor activation stimulates exocytosis directly via influx of Ca^2+^ from the extracellular space and elevation of the cytoplasmic Ca^2+^ concentration ([Ca^2+^]_c_; Kim et al., 2004; Shigetomi and Kato, 2004; Jacques-Silva et al., 2010; Hayoz et al., 2012). It is well established that a series of Ca^2+^-dependent steps during the exocytic pre-fusion stage is essential for fusion of exocytic vesicles with the plasma membrane (Burgoyne and Morgan, 1998; Sudhof, 2004; Neher and Sakaba, 2008). Ca^2+^ can either enter through P2X receptor pores themselves or through voltage-gated Ca^2+^ channels, which are activated as a consequence of the P2X receptor-mediated membrane depolarization (Khakh and North, 2006). In line with these findings, several studies proposed a role for P2X_4_ receptors in exocytosis that is mediated via an increase in the intracellular Ca^2+^ concentration. P2X_4_ receptors have a relatively slow desensitization (5–10 s) and a high Ca^2+^ permeability, Ca^2+^ contributes 8% of the whole current in human P2X_4_ (Wang et al., 1996; Garcia-Guzman et al., 1997; North, 2002; Egan and Khakh, 2004). Hence, activation of P2X_4_ receptors can generate sufficient increases in [Ca^2+^]_c_ to stimulate regulated exocytosis. Indeed, insulin secretion from pancreatic islets (Ohtani et al., 2011) and exocytic response in parotid acinar cells (Bhattacharya et al., 2012) following stimulation with ATP were augmented in the presence of ivermectin, a selective potentiator of P2X_4_ receptor currents (Khakh et al., 1999). P2X_4_ activation was also found to modulate glutamate and gamma-aminobutyric acid (GABA) release in hypothalamic neurons (Vavra et al., 2011) and brain-derived neurotrophic factor (BDNF) in microglial cells (Trang et al., 2009).

In all of these systems, activation of P2X receptors adjusts the secretory output predominantly by modulating the number of vesicles that fuse with the plasma membrane. Depending on the cell type and the shape of the Ca^2+^ signal, the rise in [Ca^2+^]_c_ triggers fusion of secretory vesicles with the plasma membrane, but also affects maturation and trafficking of secretory vesicles to the plasma membrane (Neher and Sakaba, 2008; Dolensek et al., 2011; Gutierrez-Martin et al., 2011).

## VESICULAR P2X_4_ RECEPTORS PROMOTE SURFACTANT SECRETION VIA FACE – “FUSION-ACTIVATED Ca^2+^-ENTRY”

Apart from regulating secretion via adjusting the number of fusing organelles the amount and composition of the secretory output is – at least for exocytosis of large secretory granules and secretion of bulky vesicle contents – modulated following fusion of the vesicle with the plasma membrane during the so-called exocytic “post-fusion” phase. Recent evidence also suggests a role for P2X_4_ receptors therein. It has been demonstrated that activation of P2X_4_ receptors following vesicle–plasma membrane fusion modulates the secretion and activation of pulmonary surfactant (Miklavc et al., 2011; Dietl et al., 2012; Thompson et al., 2013).

Pulmonary surfactant is secreted via exocytosis of lamellar bodies (LBs), large lysosome-related storage organelles in alveolar type II (ATII) epithelial cells. Surfactant is stored in LBs as densely packed membranous structures that do not readily diffuse out of fused LBs following opening of the exocytic fusion pore. Rather, surfactant is so insoluble, that it may remain entrapped within the fused vesicle for many minutes and the slowly expanding fusion pore acts as a mechanical barrier for the release (Dietl et al., 2001; Haller et al., 2001; Singer et al., 2003; Dietl and Haller, 2005; Miklavc et al., 2012).

**FIGURE 1:**
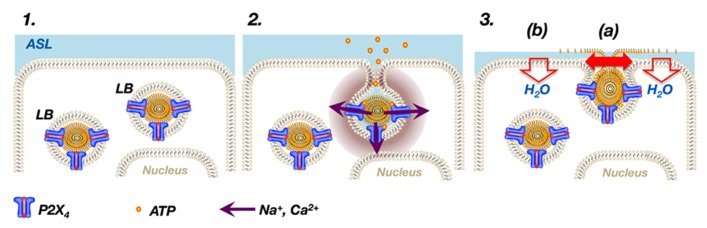
**P2X_4_ receptors on LBs modulate surfactant secretion.** P2X_4_ receptors are expressed on LBs, large storage organelles for pulmonary surfactant in ATII cells **(1)**. Upon exocytosis of LBs, P2X_4_ receptors readily become part of the apical membrane and activation of P2X_4_ by extracellular ATP results in a transient, non-selective, inward-rectifying, cation current at the site of the fused vesicle causing a local increase in Ca^2+^ around the fused vesicle **(2)**. The local increase in Ca^2+^ promotes fusion pore expansion **(3a)**. In addition, the inward-rectifying cation current on the apical side results in vectorial ion transport across ATII cells, which in turn promotes fluid resorption and thereby facilitates adsorption of newly released surfactant into the air–liquid interface **(3b)**. ASL = alveolar surface liquid.

Miklavc et al. (2010) initially discovered that exocytosis of LBs results in localized Ca^2+^ influx at the site of vesicle fusion which they termed “FACE” for “fusion-activated Ca^2+^-entry”. Subsequently, they found that FACE is mediated via activation of P2X_4_ receptors expressed on the limiting membranes of LBs (Miklavc et al., 2011). Upon exocytosis of LBs, the P2X_4_ receptor is readily part of the apical membrane as soon as membrane fusion is completed (Miklavc et al., 2009). Activation of P2X_4_ in the presence of extracellular ATP then results in a transient, non-selective, inward-rectifying, cation current at the site of the fused vesicle (Miklavc et al., 2011; Thompson et al., 2013) (**Figure 1**). The relatively high Ca^2+^ permeability of P2X_4_ receptors (North, 2002) causes a local, transient rise of [Ca^2+^]_c_ around the fused vesicle which promotes fusion pore expansion (Miklavc et al., 2011). In ATII cells, vesicle content (i.e., surfactant) release is tightly regulated via Ca^2+^-dependent fusion pore expansion (Haller et al., 2001) and it has been demonstrated that FACE via P2X_4_ receptors on LBs directly facilitates surfactant release in the alveolus (Miklavc et al., 2011).

Ca^2+^ channels localized in the membranes of the secretory vesicles that respond to changes in the membrane potential or extracellular agonists upon fusion are ideally suited for generating a localized rise in Ca^2+^ and selectively affect the individual fused vesicle. Yet, so far such mechanisms have only been known in invertebrates (Smith et al., 2000; Yao et al., 2009; Miklavc and Frick, 2011) and P2X_4_ receptors on LBs resemble the first analog mechanism in mammals. It will be interesting to see whether a similar role for P2X receptors is present in other secretory cells. Similar to LBs in ATII cells, many different cell types harbor secretory lysosomes or lysosome-related organelles to store for secretory products that are released via exocytosis of these organelles (Dell’Angelica et al., 2000; Blott and Griffiths, 2002; Luzio et al., 2007). Many of these contain rather bulky, macromolecular vesicle contents and release is modulated via the exocytic post-fusion phase (Thorn, 2009). In addition, it is well established that P2X receptors, in particular P2X_4_, are predominantly located within lysosomal compartments and inserted into the cell surface upon exocytosis (Qureshi et al., 2007; Toyomitsu et al., 2012).

## VESICULAR P2X_4_ RECEPTORS FACILITATE “ACTIVATION” OF SURFACTANT

Following release into the alveolar hypophase surfactant maintains its compact organization, constituting lamellar body-like particles (LBPs; Haller et al., 2004). To gain its vital function of reducing the surface tension within alveoli, it needs to be inserted into the air–liquid interface. Freshly released LBPs disintegrate when they contact an air–liquid interface, leading to instantaneous spreading and insertion of surfactant material at this interface (Dietl and Haller, 2005). Thompson et al. (2013) demonstrated that, in addition to facilitating fusion pore dilation, FACE via P2X_4_ also drives fluid resorption from the alveolar lumen. The P2X_4_ mediated inward-rectifying cation current on the apical side results in vectorial ion transport across ATII cells, which in turn promotes apical to basolateral fluid transport (Thompson et al., 2013) (**Figure 1**). FACE-dependent transepithelial fluid resorption is a rather transient process which requires the presence of luminal ATP or other P2X_4_ agonists and hence it is unlikely that it is a major contributor to regulation of alveolar liquid homeostasis under physiological conditions (Folkesson and Matthay, 2006). However, it has been suggested that this localized alveolar fluid resorption results in temporary thinning of the alveolar hypophase which in turn promotes contact between LBPs and the interphase and facilitates adsorption of newly released surfactant into the air–liquid interface (Thompson et al., 2013). Hence, activation of P2X_4_ and FACE (which in order to embrace the true nature of FACE should now be referred to as “fusion-activated cation entry”) facilitates surfactant release via fusion pore opening and contributes to “activation” or “functionalising” of surfactant. Such a temporal and local coordination of surfactant secretion and reduction of alveolar lining fluid could constitute a powerful mechanism for fine-tuning surfactant replenishment – the integrators being vesicular P2X_4_ receptors and extracellular ATP.

## ATP AS INTEGRATOR FOR SURFACTANT SECRETION AND “ACTIVATION”

It is intriguing that extracellular ATP plays multiple functions for surfactant secretion in the alveolus. Apart from P2X_4_ receptors, ATII cells also express P2Y_2_ receptors (Garcia-Verdugo et al., 2008; Burnstock et al., 2012) and activation thereof is one of the most potent stimuli for LB exocytosis and surfactant secretion (Rice and Singleton, 1987; Frick et al., 2001; Andreeva et al., 2007; Dietl et al., 2010). Hence, ATP is integrating the entire secretion process from stimulating LB exocytosis to facilitating surfactant release and “activating” surfactant during the post-fusion phase.

Despite this importance of ATP for lung function, the origins of ATP in the alveoli are still elusive. It has been reported that ATP is present in the pulmonary hypophase (Patel et al., 2005), however, the estimated concentration under resting conditions is in the low nM range (Bove et al., 2010), well below the EC_50_ values for P2X_4_ activation (North, 2002) or P2Y_2_ activation (Lazarowski et al., 1995; Brunschweiger and Muller, 2006).

Cell stretch during deep inflation is considered the most potent if not only physiologically relevant stimulus for surfactant secretion (Wirtz and Dobbs, 2000; Dietl et al., 2004, 2010; Frick et al., 2004) and stretch-induced ATP release from alveolar epithelial cells (Patel et al., 2005; Mishra et al., 2011) could represent a key regulatory element (Dietl et al., 2010). Several possible pathways for ATP release have been described in the respiratory epithelia. ATP can either be released into the hypophase via regulated exocytosis from secretory cells (Kreda et al., 2010; Okada et al., 2011), or in a conductive way via pannexin hemichannels (Ransford et al., 2009; Seminario-Vidal et al., 2011) or P2X_7_ receptors (Mishra et al., 2011). In particular, local ATP release within individual alveoli may provide an ideal mechanism to gradually adapt local surfactant secretion to local demands. The alveolar epithelium consists of only two cell types; besides surfactant secreting ATII cells, flat alveolar type I (ATI) cells cover most of the alveolar surface. In contrast to primary ATII cells that only express P2X_4_ receptors (Miklavc et al., 2011) ATI cells express P2X_4_ and P2X_7_ receptors (Weinhold et al., 2010; Burnstock et al., 2012). P2X_7_ knock-out mice fail to increase surfactant secretion in response to hyperventilation and substantial evidence suggests that ATP release via P2X_7_ receptors on ATI cells maintains alveolar surfactant homeostasis in response to increased alveolar distension by stimulating P2Y_2_ receptors on ATII cells (Mishra et al., 2011) and, in light of our recent findings, possible activation of P2X_4_ (Miklavc et al., 2011; Thompson et al., 2013). In addition to responding to mechanical distension of alveoli, alveolar epithelial cells also respond to increased tension forces at the air–liquid interphase with exocytic release of ATP (e.g., upon local depletion of surfactant or when coming in close proximity to the air–liquid interphase following a decrease in alveolar hypophase height; Ramsingh et al., 2011).

Whether ATII cells also release ATP, to act in an autocrine feedback loop, is still unknown. Many secretory vesicles, including lysosome-related organelles, have been found to contain significant amounts of ATP (Bodin and Burnstock, 2001; Praetorius and Leipziger, 2009; Lazarowski et al., 2011) and it has been reported that ATP is released from ATII-like A549 cells, likely via exocytosis (Tatur et al., 2008; Ramsingh et al., 2011). It is tempting to speculate that LBs contain ATP and hence provide the ligand for the P2X_4_ receptors themselves. In such a scenario, the high degree of pH sensitivity of this receptor (Clarke et al., 2000; Zsembery et al., 2003; Coddou et al., 2011) could prevent intravesicular activation of the receptor in the presence of vesicular ATP (pH of LB is <6.1; Chander et al., 1986).

Also, under pathophysiological conditions resulting from many chronic lung diseases, release of purine nucleotides from respiratory epithelia is significantly increased (Adriaensen and Timmermans, 2004; Lommatzsch et al., 2010). It has been demonstrated that trauma-induced damage of the alveolus leads to substantial ATP release and that extracellular ATP is a key player to rescue alveolar function following damage, including regulation of surfactant secretion (Riteau et al., 2010; Belete et al., 2011). In addition, several studies have demonstrated up-regulation of P2X receptors in various cell types during pathological conditions including inflammation, tumor growth, and injury (Burnstock and Kennedy, 2011) and it has been hypothesized that chronic extracellular ATP may be responsible (Geisler et al., 2013). Such a mechanism could be particularly relevant in the lung, and P2X receptors may play an even greater role in many pathological conditions with chronically increased extracellular ATP levels. Initial evidence came from studies indicating that smoke-induced lung inflammation leads to increased levels of ATP in broncho-alveolar fluid and up-regulation of P2X_7_ expression (Lommatzsch et al., 2010; Lucattelli et al., 2011). A similar role for P2X_4_ receptors under pathophysiological conditions is still to be confirmed. However, it is becoming increasingly evident that purinergic signaling is taking center stage in regulating secretion of pulmonary surfactant and adapting it to local demands under physiological and diseased conditions. P2X_4_ receptors on LBs provide an ideal mechanism for fine-tuning surfactant secretion via ATP levels in the alveolar hypophase.

Despite recent advances in our understanding how purinergic signaling in the alveolus, in particular activation of vesicular P2X_4_ receptors, modulates LB exocytosis, surfactant secretion and activation of released surfactant, several important questions still remain to be answered: What is the physiological relevance of such a complex regulatory mechanism? What are the sources of ATP under physiological and more importantly pathophysiological conditions? And – extending the scope from the lung – is purinergic signaling a general mechanism for secretion of large, macromolecular vesicle contents or is it unique to LB exocytosis and surfactant secretion? The answers to these questions warrant further research and certainly promise an increased understanding of the role of P2X receptors in regulating exocytosis and cellular secretion.

## Conflict of Interest Statement

The authors declare that the research was conducted in the absence of any commercial or financial relationships that could be construed as a potential conflict of interest.

## References

[B1] Adriaensen D. Timmermans J. P. (2004). Purinergic signalling in the lung: important in asthma and COPD? *Curr. Opin. Pharmacol.* 4 207–214 10.1016/j.coph.2004.01.010 15140410

[B2] Andreeva A. V. Kutuzov M. A. Voyno-Yasenetskaya T. A. (2007). Regulation of surfactant secretion in alveolar type II cells. *Am. J. Physiol. Lung Cell. Mol. Physiol.* 293 L259–L271 10.1152/ajplung.00112.2007 17496061

[B3] Belete H. A. Hubmayr R. D. Wang S. Singh R. D. (2011). The role of purinergic signaling on deformation induced injury and repair responses of alveolar epithelial cells. *PLoS ONE* 6:e27469 10.1371/journal.pone.0027469 PMC3210789 22087324

[B4] Bhattacharya S. Verrill D. S. Carbone K. M. Brown S. Yule D. I Giovannucci D. R. (2012). Distinct contributions by ionotropic purinoceptor subtypes to ATP-evoked calcium signals in mouse parotid acinar cells. *J. Physiol.* 590 2721–2737 10.1113/jphysiol.2012.228148 22451435 PMC3424727

[B5] Blott E. J. Griffiths G. M. (2002). Secretory lysosomes. *Nat. Rev. Mol. Cell Biol.* 3 122–131 10.1038/nrm732 11836514

[B6] Bodin P. Burnstock G. (2001). Purinergic signalling: ATP release. *Neurochem. Res.* 26 959–969 10.1023/A:1012388618693 11699948

[B7] Bove P. F. Grubb B. R. Okada S. F. Ribeiro C. M. Rogers T. D. Randell S. H. (2010). Human alveolar type II cells secrete and absorb liquid in response to local nucleotide signaling. *J. Biol. Chem.* 285 34939–34949 10.1074/jbc.M110.162933 20801871 PMC2966108

[B8] Brake A. J. Wagenbach M. J. Julius D. (1994). New structural motif for ligand-gated ion channels defined by an ionotropic ATP receptor. *Nature* 371 519–523 10.1038/371519a0 7523952

[B9] Brunschweiger A. Muller C. E. (2006). P2 receptors activated by uracil nucleotides – an update. *Curr. Med. Chem.* 13 289–312 10.2174/092986706775476052 16475938

[B10] Burgoyne R. D. Morgan A. (1998). Calcium sensors in regulated exocytosis. *Cell Calcium* 24 367–376 10.1016/S0143-4160(98)90060-4 10091006

[B11] Burnstock G. Brouns I. Adriaensen D. Timmermans J. P. (2012). Purinergic signaling in the airways. *Pharmacol. Rev.* 64 834–868 10.1124/pr.111.005389 22885703

[B12] Burnstock G. Kennedy C. (2011). P2X receptors in health and disease. *Adv. Pharmacol.* 61 333–372 10.1016/B978-0-12-385526-8.00011-4 21586364

[B13] Chander A. Johnson R. G. Reicherter J. Fisher A. B. (1986). Lung lamellar bodies maintain an acidic internal pH. *J. Biol. Chem.* 261 6126–6131 3700387

[B14] Clarke C. E. Benham C. D. Bridges A. George A. R. Meadows H. J. (2000). Mutation of histidine 286 of the human P2X_4_ purinoceptor removes extracellular pH sensitivity. *J. Physiol.* 523(Pt 3) 697–703 10.1111/j.1469-7793.2000.00697.x 10718748 PMC2269823

[B15] Cockcroft S. Gomperts B. D. (1979a). Activation and inhibition of calcium-dependent histamine secretion by ATP ions applied to rat mast cells. *J. Physiol.* 296 229–243 93638 10.1113/jphysiol.1979.sp013002 PMC1279075

[B16] Cockcroft S. Gomperts B. D. (1979b). ATP induces nucleotide permeability in rat mast cells. *Nature* 279 541–542 10.1038/279541a0 450099

[B17] Cockcroft S. Gomperts B. D. (1980). The ATP4-receptor of rat mast cells. *Biochem. J.* 188 789–798 6162453 10.1042/bj1880789 PMC1161963

[B18] Coddou C. Yan Z. Obsil T. Huidobro-Toro J. P. Stojilkovic S. S. (2011). Activation and regulation of purinergic P2X receptor channels. *Pharmacol. Rev.* 63 641–683 10.1124/pr.110.003129 21737531 PMC3141880

[B19] Dell’Angelica E. C. Mullins C. Caplan S. Bonifacino J. S. (2000). Lysosome-related organelles. *FASEB J.* 14 1265–1278 10.1096/fj.14.10.1265 10877819

[B20] Diamant B. Kruger P. G. (1967). Histamine release from isolated rat peritoneal mast cells induced by adenosine-5′-triphosphate. *Acta Physiol. Scand.* 71 291–302 10.1111/j.1748-1716.1967.tb03736.x 4172485

[B21] Dietl P. Frick M. Mair N. Bertocchi C. Haller T. (2004). Pulmonary consequences of a deep breath revisited. *Biol. Neonate* 85 299–304 10.1159/000078176 15218287

[B22] Dietl P. Haller T. (2005). Exocytosis of lung surfactant: from the secretory vesicle to the air–liquid interface. *Annu. Rev. Physiol.* 67 595–621 10.1146/annurev.physiol.67.040403.102553 15709972

[B23] Dietl P. Haller T. Frick M. (2012). Spatio-temporal aspects, pathways and actions of Ca(^2+^) in surfactant secreting pulmonary alveolar type II pneumocytes. *Cell Calcium* 52 296–302 10.1016/j.ceca.2012.04.010 22591642

[B24] Dietl P. Haller T. Mair N. Frick M. (2001). Mechanisms of surfactant exocytosis in alveolar type II cells *in vitro* and *in vivo*. *News Physiol. Sci.* 16 239–243 11572929 10.1152/physiologyonline.2001.16.5.239

[B25] Dietl P. Liss B. Felder E. Miklavc P. Wirtz H. (2010). Lamellar body exocytosis by cell stretch or purinergic stimulation: possible physiological roles, messengers and mechanisms. *Cell. Physiol. Biochem.* 25 1–12 10.1159/000272046 20054140

[B26] Dolensek J. Skelin M. Rupnik M. S. (2011). Calcium dependencies of regulated exocytosis in different endocrine cells. *Physiol. Res.* 60(Suppl. 1) S29–S38 21777026 10.33549/physiolres.932176

[B27] Egan T. M. Khakh B. S. (2004). Contribution of calcium ions to P2X channel responses. *J. Neurosci.* 24 3413–3420 10.1523/JNEUROSCI.5429-03.2004 15056721 PMC6730036

[B28] Folkesson H. G. Matthay M. A. (2006). Alveolar epithelial ion and fluid transport: recent progress. *Am. J. Respir. Cell Mol. Biol.* 35 10–19 10.1165/rcmb.2006-0080SF 16514116 PMC2658691

[B29] Frick M. Bertocchi C. Jennings P. Haller T. Mair N. Singer W. (2004). Ca^2+^ entry is essential for cell strain-induced lamellar body fusion in isolated rat type II pneumocytes. *Am. J. Physiol. Lung Cell. Mol. Physiol.* 286 L210–L220 10.1152/ajplung.00332.2003 14504067

[B30] Frick M. Eschertzhuber S. Haller T. Mair N. Dietl P. (2001). Secretion in alveolar type II cells at the interface of constitutive and regulated exocytosis. *Am. J. Respir. Cell Mol. Biol.* 25 306–315 10.1165/ajrcmb.25.3.4493 11588008

[B31] Garcia-Guzman M. Soto F. Gomez-Hernandez J. M. Lund P. E. Stuhmer W. (1997). Characterization of recombinant human P2X_4_ receptor reveals pharmacological differences to the rat homologue. *Mol. Pharmacol.* 51 109–118 9016352 10.1124/mol.51.1.109

[B32] Garcia-Verdugo I. Ravasio A. De Paco E. G. Synguelakis M. Ivanova N. Kanellopoulos J. (2008). Long-term exposure to LPS enhances the rate of stimulated exocytosis and surfactant secretion in alveolar type II cells and upregulates P2Y2 receptor expression. *Am. J. Physiol. Lung Cell. Mol. Physiol.* 295 L708–L717 10.1152/ajplung.00536.2007 18689605

[B33] Geisler J. C. Corbin K. L. Li Q. Feranchak A. P. Nunemaker C. S. Li C. (2013). Vesicular nucleotide transporter-mediated ATP release regulates insulin secretion. *Endocrinology* 154 675–684 10.1210/en.2012-1818 23254199 PMC3548185

[B34] Gu J. G. MacDermott A. B. (1997). Activation of ATP P2X receptors elicits glutamate release from sensory neuron synapses. *Nature* 389 749–753 10.1038/39639 9338789

[B35] Gutierrez-Martin Y. Bustillo D. Gomez-Villafuertes R. Sanchez-Nogueiro J. Torregrosa-Hetland C. Binz T. (2011). P2X7 receptors trigger ATP exocytosis and modify secretory vesicle dynamics in neuroblastoma cells. *J. Biol. Chem.* 286 11370–11381 10.1074/jbc.M110.139410 21292765 PMC3064193

[B36] Haller T. Dietl P. Pfaller K. Frick M. Mair N. Paulmichl M. (2001). Fusion pore expansion is a slow, discontinuous, and Ca^2+^-dependent process regulating secretion from alveolar type II cells. *J. Cell Biol.* 155 279–289 10.1083/jcb.200102106 11604423 PMC2198834

[B37] Haller T. Dietl P. Stockner H. Frick M. Mair N. Tinhofer I. (2004). Tracing surfactant transformation from cellular release to insertion into an air–liquid interface. *Am. J. Physiol. Lung Cell. Mol. Physiol.* 286 L1009–L1015 10.1152/ajplung.00342.2003 14704221

[B38] Hayoz S. Jia C. Hegg C. (2012). Mechanisms of constitutive and ATP-evoked ATP release in neonatal mouse olfactory epithelium. *BMC Neurosci.* 13:53 10.1186/1471-2202-13-53 PMC3444318 22640172

[B39] Huang Y. A. Stone L. M. Pereira E. Yang R. Kinnamon J. C. Dvoryanchikov G. (2011). Knocking out P2X receptors reduces transmitter secretion in taste buds. *J. Neurosci.* 31 13654–13661 10.1523/JNEUROSCI.3356-11.2011 21940456 PMC3188419

[B40] Jacques-Silva M. C. Correa-Medina M. Cabrera O. Rodriguez-Diaz R. Makeeva N. Fachado A. (2010). ATP-gated P2X3 receptors constitute a positive autocrine signal for insulin release in the human pancreatic beta cell. *Proc. Natl. Acad. Sci. U.S.A.* 107 6465–6470 10.1073/pnas.0908935107 20308565 PMC2851966

[B41] Kaczmarek-Hajek K. Lorinczi E. Hausmann R. Nicke A. (2012). Molecular and functional properties of P2X receptors – recent progress and persisting challenges. *Purinergic Signal.* 8 375–417 10.1007/s11302-012-9314-7 22547202 PMC3360091

[B42] Khakh B. S. North R. A. (2006). P2X receptors as cell-surface ATP sensors in health and disease. *Nature* 442 527–532 10.1038/nature04886 16885977

[B43] Khakh B. S. Proctor W. R. Dunwiddie T. V. Labarca C. Lester H. A. (1999). Allosteric control of gating and kinetics at P2X(_4_) receptor channels. *J. Neurosci.* 19 7289–7299 10460235 10.1523/JNEUROSCI.19-17-07289.1999 PMC6782529

[B44] Kim J. H. Nam J. H. Kim M. H. Koh D. S. Choi S. J. Kim S. J. (2004). Purinergic receptors coupled to intracellular Ca^2+^ signals and exocytosis in rat prostate neuroendocrine cells. *J. Biol. Chem.* 279 27345–27356 10.1074/jbc.M313575200 15100230

[B45] Kreda S. M. Seminario-Vidal L. Van Heusden C. A. O’Neal W. Jones L. Boucher R. C. (2010). Receptor-promoted exocytosis of airway epithelial mucin granules containing a spectrum of adenine nucleotides. *J. Physiol.* 588 2255–2267 10.1113/jphysiol.2009.186643 20421285 PMC2911224

[B46] Lazarowski E. R. Sesma J. I. Seminario-Vidal L. Kreda S. M. (2011). Molecular mechanisms of purine and pyrimidine nucleotide release. *Adv. Pharmacol.* 61 221–261 10.1016/B978-0-12-385526-8.00008-4 21586361

[B47] Lazarowski E. R. Watt W. C. Stutts M. J. Boucher R. C. Harden T. K. (1995). Pharmacological selectivity of the cloned human P2U-purinoceptor: potent activation by diadenosine tetraphosphate. *Br. J. Pharmacol.* 116 1619–1627 10.1111/j.1476-5381.1995.tb16382.x 8564228 PMC1908898

[B48] Lommatzsch M. Cicko S. Muller T. Lucattelli M. Bratke K. Stoll P. (2010). Extracellular adenosine triphosphate and chronic obstructive pulmonary disease. *Am. J. Respir. Crit. Care Med.* 181 928–934 10.1164/rccm.200910-1506OC 20093639

[B49] Lucattelli M. Cicko S. Muller T. Lommatzsch M. De Cunto G. Cardini S. (2011). P2X7 receptor signaling in the pathogenesis of smoke-induced lung inflammation and emphysema. *Am. J. Respir. Cell Mol. Biol.* 44 423–429 10.1165/rcmb.2010-0038OC 20508069

[B50] Luzio J. P. Pryor P. R. Bright N. A. (2007). Lysosomes: fusion and function. *Nat. Rev. Mol. Cell Biol.* 8 622–632 10.1038/nrm2217 17637737

[B51] MacKenzie A. Wilson H. L. Kiss-Toth E. Dower S. K. North R. A. Surprenant A. (2001). Rapid secretion of interleukin-1beta by microvesicle shedding. *Immunity* 15 825–835 10.1016/S1074-7613(01)00229-1 11728343

[B52] Miklavc P. Albrecht S. Wittekindt O. H. Schullian P. Haller T. Dietl P. (2009). Existence of exocytotic hemifusion intermediates with a lifetime of up to seconds in type II pneumocytes. *Biochem. J.* 424 7–14 10.1042/BJ20091094 19712048

[B53] Miklavc P. Frick M. (2011). Vesicular calcium channels as regulators of the exocytotic post-fusion phase. *Commun. Integr. Biol.* 4 796–798 22446559 10.4161/cib.17935 PMC3306363

[B54] Miklavc P. Frick M. Wittekindt O. H. Haller T. Dietl P. (2010). Fusion-activated Ca(^2+^) entry: an “active zone” of elevated Ca(^2+^) during the post-fusion stage of lamellar body exocytosis in rat type II pneumocytes. *PLoS ONE* 5:e10982 10.1371/journal.pone.0010982 PMC2882333 20544027

[B55] Miklavc P. Hecht E. Hobi N. Wittekindt O. H. Dietl P. Kranz C. (2012). Actin coating and compression of fused secretory vesicles are essential for surfactant secretion~– a role for Rho, formins and myosin II. *J. Cell Sci.* 125 2765–2774 10.1242/jcs.105262 22427691

[B56] Miklavc P. Mair N. Wittekindt O. H. Haller T. Dietl P. Felder E. (2011). Fusion-activated Ca^2+^ entry via vesicular P2X_4_ receptors promotes fusion pore opening and exocytotic content release in pneumocytes. *Proc. Natl. Acad. Sci. U.S.A.* 108 14503–14508 10.1073/pnas.1101039108 21844344 PMC3167537

[B57] Mishra A. Chintagari N. R. Guo Y. Weng T. Su L. Liu L. (2011). Purinergic P2X7 receptor regulates lung surfactant secretion in a paracrine manner. *J. Cell Sci.* 124 657–668 10.1242/jcs.066977 21266468 PMC3031375

[B58] Neher E. Sakaba T. (2008). Multiple roles of calcium ions in the regulation of neurotransmitter release. *Neuron* 59 861–872 10.1016/j.neuron.2008.08.019 18817727

[B59] North R. A. (2002). Molecular physiology of P2X receptors. *Physiol. Rev.* 82 1013–1067 12270951 10.1152/physrev.00015.2002

[B60] Novak I. (2011). Purinergic signalling in epithelial ion transport: regulation of secretion and absorption. *Acta Physiol. (Oxf.)* 202 501–522 10.1111/j.1748-1716.2010.02225.x 21073662

[B61] Ohtani M. Ohura K. Oka T. (2011). Involvement of P2X receptors in the regulation of insulin secretion, proliferation and survival in mouse pancreatic beta-cells. *Cell. Physiol. Biochem.* 28 355–366 10.1159/000331752 21865744

[B62] Okada S. F. Zhang L. Kreda S. M. Abdullah L. H. Davis C. W. Pickles R. J. (2011). Coupled nucleotide and mucin hypersecretion from goblet-cell metaplastic human airway epithelium. *Am. J. Respir. Cell Mol. Biol.* 45 253–260 10.1165/rcmb.2010-0253OC 20935191 PMC3175555

[B63] Patel A. S. Reigada D. Mitchell C. H. Bates S. R. Margulies S. S. Koval M. (2005). Paracrine stimulation of surfactant secretion by extracellular ATP in response to mechanical deformation. *Am. J. Physiol. Lung Cell. Mol. Physiol.* 289 L489–L496 10.1152/ajplung.00074.2005 15908478

[B64] Praetorius H. A. Leipziger J. (2009). ATP release from non-excitable cells. *Purinergic Signal.* 5 433–446 10.1007/s11302-009-9146-2 19301146 PMC2776134

[B65] Qu Y. Dubyak G. R. (2009). P2X7 receptors regulate multiple types of membrane trafficking responses and non-classical secretion pathways. *Purinergic Signal.* 5 163–173 10.1007/s11302-009-9132-8 19189228 PMC2686822

[B66] Qu Y. Franchi L. Nunez G. Dubyak G. R. (2007). Nonclassical IL-1 beta secretion stimulated by P2X7 receptors is dependent on inflammasome activation and correlated with exosome release in murine macrophages. *J. Immunol.* 179 1913–1925 17641058 10.4049/jimmunol.179.3.1913

[B67] Qureshi O. S. Paramasivam A. Yu J. C. Murrell-Lagnado R. D. (2007). Regulation of P2X_4_ receptors by lysosomal targeting, glycan protection and exocytosis. *J. Cell Sci.* 120 3838–3849 10.1242/jcs.010348 17940064

[B68] Ramsingh R. Grygorczyk A. Solecki A. Cherkaoui L. S. Berthiaume Y. Grygorczyk R. (2011). Cell deformation at the air-liquid interface induces Ca^2+^-dependent ATP release from lung epithelial cells. *Am. J. Physiol. Lung Cell. Mol. Physiol.* 300 L587–L595 10.1152/ajplung.00345.2010 21239538

[B69] Ransford G. A. Fregien N. Qiu F. Dahl G. Conner G. E. Salathe M. (2009). Pannexin 1 contributes to ATP release in airway epithelia. *Am. J. Respir. Cell Mol. Biol.* 41 525–534 10.1165/rcmb.2008-0367OC 19213873 PMC2778159

[B70] Rice W. R. Singleton F. M. (1987). P2Y-purinoceptor regulation of surfactant secretion from rat isolated alveolar type II cells is associated with mobilization of intracellular calcium. *Br. J. Pharmacol.* 91 833–838 10.1111/j.1476-5381.1987.tb11282.x 3664080 PMC1853589

[B71] Riteau N. Gasse P. Fauconnier L. Gombault A. Couegnat M. Fick L. (2010). Extracellular ATP is a danger signal activating P2X7 receptor in lung inflammation and fibrosis. *Am. J. Respir. Crit. Care Med.* 182 774–783 10.1164/rccm.201003-0359OC 20522787

[B72] Rodriguez Candela J. L. Garcia-Fernandez M. C. (1963). Stimulation of secretion of insulin by adenosine-triphosphate. *Nature* 197 1210 10.1038/1971210a0 13990873

[B73] Seminario-Vidal L. Okada S. F. Sesma J. I. Kreda S. M. Van Heusden C. A. Zhu Y. (2011). Rho signaling regulates pannexin 1-mediated ATP release from airway epithelia. *J. Biol. Chem.* 286 26277–26286 10.1074/jbc.M111.260562 21606493 PMC3143590

[B74] Shigetomi E. Kato F. (2004). Action potential-independent release of glutamate by Ca^2+^ entry through presynaptic P2X receptors elicits postsynaptic firing in the brainstem autonomic network. *J. Neurosci.* 24 3125–3135 10.1523/JNEUROSCI.0090-04.2004 15044552 PMC6729830

[B75] Singer W. Frick M. Haller T. Bernet S. Ritsch-Marte M. Dietl P. (2003). Mechanical forces impeding exocytotic surfactant release revealed by optical tweezers. *Biophys. J.* 84 1344–1351 10.1016/S0006-3495(03)74950-9 12547815 PMC1302711

[B76] Smith R. M. Baibakov B. Ikebuchi Y. White B. H. Lambert N. A. Kaczmarek L. K. (2000). Exocytotic insertion of calcium channels constrains compensatory endocytosis to sites of exocytosis. *J. Cell Biol.* 148 755–767 10.1083/jcb.148.4.755 10684256 PMC2169375

[B77] Solini A. Chiozzi P. Morelli A. Fellin R Di Virgilio F. (1999). Human primary fibroblasts *in vitro* express a purinergic P2X7 receptor coupled to ion fluxes, microvesicle formation and IL-6 release. *J. Cell Sci.* 112(Pt 3) 297–305 9885283 10.1242/jcs.112.3.297

[B78] Sudhof T. C. (2004). The synaptic vesicle cycle. *Annu. Rev. Neurosci.* 27 509–547 10.1146/annurev.neuro.26.041002.131412 15217342

[B79] Surprenant A. (1996). Functional properties of native and cloned P2X receptors. *Ciba Found. Symp.* 198 208–219; discussion 219–222 8879827 10.1002/9780470514900.ch12

[B80] Surprenant A. Rassendren F. Kawashima E. North R. A. Buell G. (1996). The cytolytic P2Z receptor for extracellular ATP identified as a P2X receptor (P2X7). *Science* 272 735–738 10.1126/science.272.5262.735 8614837

[B81] Tatur S. Kreda S. Lazarowski E. Grygorczyk R. (2008). Calcium-dependent release of adenosine and uridine nucleotides from A549 cells. *Purinergic Signal.* 4 139–146 10.1007/s11302-007-9059-x 18368524 PMC2377317

[B82] Thompson K. E. Korbmacher J. P. Hecht E. Hobi N. Wittekindt O. H. Dietl P. (2013). Fusion-activated cation entry (FACE) via P2X(_4_) couples surfactant secretion and alveolar fluid transport. *FASEB J.* 27 1772–1783 10.1096/fj.12-220533 23307836

[B83] Thorn P. (2009). New insights into the control of secretion. *Commun. Integr. Biol.* 2 315–317 10.4161/cib.2.4.8262 19721876 PMC2734033

[B84] Toyomitsu E. Tsuda M. Yamashita T. Tozaki-Saitoh H. Tanaka Y. Inoue K. (2012). CCL_2_ promotes P2X_4_ receptor trafficking to the cell surface of microglia. *Purinergic Signal.* 8 301–310 10.1007/s11302-011-9288-x 22222817 PMC3350584

[B85] Trang T. Beggs S. Wan X. Salter M. W. (2009). P2X_4_-receptor-mediated synthesis and release of brain-derived neurotrophic factor in microglia is dependent on calcium and p38-mitogen-activated protein kinase activation. *J. Neurosci.* 29 3518–3528 10.1523/JNEUROSCI.5714-08.2009 19295157 PMC3589565

[B86] Ulmann L. Hatcher J. P. Hughes J. P. Chaumont S. Green P. J. Conquet F. (2008). Up-regulation of P2X_4_ receptors in spinal microglia after peripheral nerve injury mediates BDNF release and neuropathic pain. *J. Neurosci.* 28 11263–11268 10.1523/JNEUROSCI.2308-08.2008 18971468 PMC6671487

[B87] Valera S. Hussy N. Evans R. J. Adami N. North R. A. Surprenant A. (1994). A new class of ligand-gated ion channel defined by P2x receptor for extracellular ATP. *Nature* 371 516–519 10.1038/371516a0 7523951

[B88] Vavra V. Bhattacharya A. Zemkova H. (2011). Facilitation of glutamate and GABA release by P2X receptor activation in supraoptic neurons from freshly isolated rat brain slices. *Neuroscience* 188 1–12 10.1016/j.neuroscience.2011.04.067 21575687

[B89] Wang C. Z. Namba N. Gonoi T. Inagaki N. Seino S. (1996). Cloning and pharmacological characterization of a fourth P2X receptor subtype widely expressed in brain and peripheral tissues including various endocrine tissues. *Biochem. Biophys. Res. Commun.* 220 196–202 10.1006/bbrc.1996.0380 8602843

[B90] Weinhold K. Krause-Buchholz U. Rodel G. Kasper M. Barth K. (2010). Interaction and interrelation of P2X_7_ and P2X_4_ receptor complexes in mouse lung epithelial cells. *Cell. Mol. Life Sci.* 67 2631–2642 10.1007/s00018-010-0355-1 20405163 PMC11115700

[B91] Wirtz H. R. Dobbs L. G. (2000). The effects of mechanical forces on lung functions. *Respir. Physiol.* 119 1–17 10.1016/S0034-5687(99)00092-4 10701703

[B92] Yao C. K. Lin Y. Q. Ly C. V. Ohyama T. Haueter C. M. Moiseenkova-Bell V. Y. (2009). A synaptic vesicle-associated Ca^2+^ channel promotes endocytosis and couples exocytosis to endocytosis. *Cell* 138 947–960 10.1016/j.cell.2009.06.033 19737521 PMC2749961

[B93] Zsembery A. Boyce A. T. Liang L. Peti-Peterdi J. Bell P. D. Schwiebert E. M. (2003). Sustained calcium entry through P2X nucleotide receptor channels in human airway epithelial cells. *J. Biol. Chem.* 278 13398–13408 10.1074/jbc.M212277200 12566439

